# Supplementary material for: Efficacy of Submicron Dispersible Free Phytosterols on Non-Alcoholic Fatty Liver Disease: A Pilot Study
Source: J Clin Med. 2023 Jan 27;12(3):979. doi: 10.3390/jcm12030979 (PMC9918217; doi:10.3390/jcm12030979)
Supplement: Supplementary file 1 [file jcm-12-00979-s001.zip › jcm-2111692-supplementary.pdf]

Journal of Clinical Medicine

MDPI

Supplementary Material

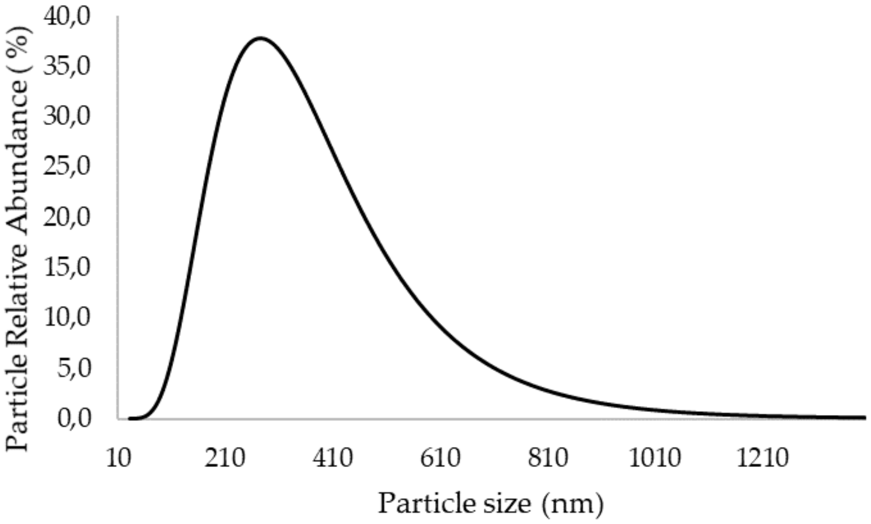

Figure S1. Size distribution of SDP. Particle size was determined by dynamic light scattering. Average size is 300 nm, and percentiles 50, 75 and 90 correspond to 270, 370 and 500 nm, respectively.

Table S1. Evaluation of fibrosis predictive scores before and after SDP treatment.

Fibrosis	NPV (%)		PPV (%)		Undetermined Zone	
Score	Base line	End point	Base line	End point	Base line	End point
APRI	96.2	96.2	0	3.85	3.85	0
HFS	100	95.8	0	4.2	NA	NA
NIS	60	36	0	8	40	56
FIB-4	84.6	80.8	0	3.8	15.4	15.4
BARD	34.6	15.4	65.4	85.6	NA	NA

AST to platelets ratio index (APRI), HepatoScore, hepamet fibrosis score (HFS), NAFLD fibrosis score (NIS), fibrosis-4 index (FIB-4) and BARD score algorithms were used for predicting fibrosis level [28], at baseline and end point after SDP treatment. Negative- and Positive-Predictive Values, NPV and PPV respectively, were determined as percentages of the studied group. Undetermined zone corresponds to subjects that do not apply to NPV nor PPV. $N = 26$ except for HFS ($n = 24$) and NIS ($n = 25$). NA, not applicable.
